# RACIAL DIFFERENCES IN ASSOCIATION OF OBJECTIVELY MEASURED PHYSICAL ACTIVITY WITH INDOOR AND OUTDOOR FALLS

**DOI:** 10.1093/geroni/igad104.0783

**Published:** 2023-12-21

**Authors:** Qun Le, Linda Churchill, Kevin Kane, Lingming Chen, Scott Crouter, Sarah Berry, Marian Hannan, Wenjun Li

**Affiliations:** University of Massachusetts Lowell, Lowell, Massachusetts, United States; University of Massachusetts, Lowell, Lowell, Massachusetts, United States; University of Massachusetts Lowell, Lowell, Massachusetts, United States; University of Massachusetts Lowell, Lowell, Massachusetts, United States; The University of Tennessee Knoxville, Knoxville, Tennessee, United States; Hebrew Rehabilitation Center, Boston, Massachusetts, United States; Harvard Medical School, Boston, Massachusetts, United States; University of Massachusetts Lowell, Lowell, Massachusetts, United States

## Abstract

Fall-related injury and hospitalization rates have been steadily increasing by 2% and 4% per year. Physical Activity Guidelines advise older adults to remain physically active to promote muscle strength, bone health, and balance. The Healthy Aging and Neighborhood Study enrolled 379 community-dwelling persons aged 65 years or older in Central Massachusetts (2018-2020). Participant physical activity (PA) levels were measured using waist-worn Actigraphy accelerometers for at least 8 hours per day for 5 or more days including 1 weekend day. Average daily PA intensities were categorized according to minutes of sedentary (SB), light (LPA), and moderate-to-vigorous-intensity PA (MVPA) using Copeland cutoffs. Falls, including the circumstances of the fall, were ascertained using monthly falls calendars and follow-up telephone interviews. Negative Binomial Regressions were used to estimate the associations of PA with rates of indoor and outdoor falls. Models were run overall and by race, with all models adjusting for age and gender. Mean age was 73.71(SD:6.41) years, 58.31%(n=221) were female, and 35.62%(n=135) were non-White. There was no significant difference in SB, LPA, and MVPA by race. White race was associated with higher rates of outdoor falls (RR(95% CI)=2.73 (1.51, 4.93)). Every 30 more minutes of MVPA was significantly associated with 25% lower rate of indoor falls (RR(95% CI)=0.74 (0.58, 0.97)) and 42% higher rate of outdoor falls (RR(95% CI)=1.42 (1.04, 1.95)) among non-White but not White (p for interaction=0.03, 0.07, respectively). Socioeconomic factors may explain the differences observed by race. Thus, future investigations should consider socioeconomic when examining indoor and outdoor falls.

